# A Framework for Annotating Human Genome in Disease Context

**DOI:** 10.1371/journal.pone.0049686

**Published:** 2012-12-10

**Authors:** Wei Xu, Huisong Wang, Wenqing Cheng, Dong Fu, Tian Xia, Warren A. Kibbe, Simon M. Lin

**Affiliations:** 1 The Department of Electronics and Information Engineering, Huazhong University of Science and Technology, Wuhan, Hubei, People's Republic of China; 2 Northwestern University Biomedical Informatics Center (NUBIC), NUCATS, Feinberg School of Medicine, Northwestern University, Chicago, Illinois, United States of America; 3 The Robert H. Lurie Comprehensive Cancer Center, Northwestern University, Chicago, Illinois, United States of America; 4 Biomedical Informatics Research Center, Marshfield Center, Marshfield, Wisconsin, United States of America; King Abdullah University of Science and Technology, Saudi Arabia

## Abstract

Identification of gene-disease association is crucial to understanding disease mechanism. A rapid increase in biomedical literatures, led by advances of genome-scale technologies, poses challenge for manually-curated-based annotation databases to characterize gene-disease associations effectively and timely. We propose an automatic method-The Disease Ontology Annotation Framework (DOAF) to provide a comprehensive annotation of the human genome using the computable Disease Ontology (DO), the NCBO Annotator service and NCBI Gene Reference Into Function (GeneRIF). DOAF can keep the resulting knowledgebase current by periodically executing automatic pipeline to re-annotate the human genome using the latest DO and GeneRIF releases at any frequency such as daily or monthly. Further, DOAF provides a computable and programmable environment which enables large-scale and integrative analysis by working with external analytic software or online service platforms. A user-friendly web interface (doa.nubic.northwestern.edu) is implemented to allow users to efficiently query, download, and view disease annotations and the underlying evidences.

## Introduction

Understanding the association between diseases and their causal genes is one of the most imperatives of biomedical research. Great effort has been made to identify gene-disease association [1], [2] and there are several database resources in practice which are dedicated to collecting the annotation of the human genome from a disease perspective, such as Online Mendelian Inheritance in Man (OMIM) [3], Genetic Association Database (GAD) [4] and Orphanet [5]. Each of databases focuses on different aspects of genetic association with diseases. For example, OMIM primarily focuses on genetic diseases with classic Mendelian inheritance; Orphanet is dedicated to information on rare diseases. These provide invaluable resources for understanding disease mechanism and developing novel approaches for prevention and treatment of the diseases.

However, the exponential growth in biomedical literatures led by the advances in DNA sequencing and related high throughput technologies poses great challenge for current annotation databases to comprehensively characterize gene-disease relationships in free-text publications/documents and keep our knowledge current. For example, the size of PubMed database reaches 22 million citations and is increasing at a rate of up to 900,000 abstracts per year [6]. On the other hand, because the OMIM is based on manual curation it usually costs time for OMIM database before updates are integrated in the database. Therefore, the coverage of knowledge bases is incomplete, given that ever-expanding repository of biomedical literature. Further, current annotation resources are mostly based on Human-readable free text but non-computable structure, which hinders direct computational consumption of the aggregate knowledgebase. In particular, they lack a controlled vocabulary which defines relationships between terms and includes consistent annotations of synonyms. For instance, “cystic renal dysplasia”, “renal dysplasia cystic,” and “renal cystic dysplasia” describe overlapping concepts in OMIM but they cannot be easily differentiated by computational analytic tool as synonyms. The widely usage of negation terms in OMIM also hampers the computational similarity calculation between two syndromes when they share a negation phrase but refer to different diseases.

To address these issues we have proposed the Disease Ontology Annotation Framework (DOAF) which integrates the Disease Ontology (DO), NCBI GeneRIF and the National Center for Bio-medical Ontology (NCBO) Annotator text mining server [7]. DOAF supports three main improvements over existing sources of gene-disease mapping: (i) DOAF provides comprehensive human disease-to-gene annotations in a computable and flexible system architecture. The disease ontology-based annotation facilitates semantic interoperability between disease terms and concepts enrichment test. (ii) Further, DOAF allows fully automatic re-annotation of the human genome at any frequency such as daily or monthly and therefore can keep our knowledge current and comprehensive. DOAF can offers a larger coverage in terms of human disease and gene-disease relationships (DOAF currently has 8,686 DO terms and 152,357 disease-gene association entries, compared to GAD's 84,000 records and OMIM's 21,382 entries where single entry may contain several disease-gene associations); (iii) The modular system architecture of DOAF is not only easy to maintain but also to facilitate integration with external analytic software or online service platforms and extension of annotating disease-gene relationships to additional species.

## Results

**Figure 1:**
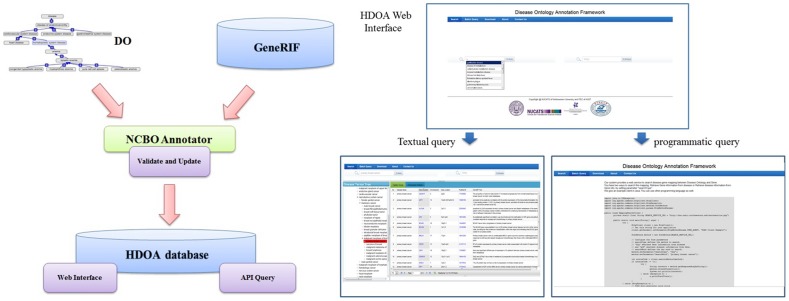
DOAF system architecture.

DOAF integrates four core functions (see **Figure 1**) - DO, GeneRIF, the NCBO Annotator, and the search interface. Organized in a single structure for classification and unification of disease and disease related concepts and terms, DO is the first ontology built around the concept of disease, although there are many vocabularies dedicated to systematically recording mortality and morbidity classifications and to standardizing clinical and event healthcare reporting [8]. DO provides a community-driven and community-accepted ontology to annotate the human genome in the context of diseases. DO, like the groundbreaking Gene Ontology (GO) [9], is organized as a directed acyclic graph and is part of the Open Biomedical Ontologies. In the DO graph, nodes are disease terms that have been manually inspected and edges capture the relationships between the terms. This semantic structure enables ontology-driven query of the annotations. In addition, DO provides cross-references to other biomedical vocabularies/ontologies including the Unified Medical Language System (UMLS) [10], the Medical Subject Headings (MeSH), Systematized Nomenclature of Medicine (SNOMED) [11], OMIM, and International Classification of Diseases ICD -9 and ICD-10.

GeneRIF contains brief textual descriptions of genes (up to 250 characters) and are available from the NCBI [12], [13]. Every GeneRIF entry is associated with a PubMed ID, providing published evidence for each description. It has been documented that abstracts and titles provided by MEDLINE are used as sources for many biomedical text mining applications [14], [15]. However, it shows difficulties to precisely identify disease-gene relationship due to free text format of MEDLINE abstracts and titles. For example, the usage of gene names suffer from ambiguity and may show different meanings which only depends on the context around them. Further, a variety of synonyms, acronyms, and abbreviations of genes or other biomedical terms are hurdles for precise semantic mining (please refer to [16] for details). GeneRIF provides a solution to these issues with manually curated deterministic gene-relevant association information in concise and simple format, although GeneRIF contain less information compared to MEDLINE abstracts and titles. A GeneRIF contains gene-disease association and any disease described should be correctly mapped to its corresponding gene unless curator incorrectly associated the gene with the disease in the GeneRIF. GeneRIF has proven to be an excellent source of evidence for identifying p53-affected genes, annotating human genome, and mapping genes to disease. Further, it is very easy to maintain due to lightweight text [17]–[19].

To link genes with disease information, the DOAF proposes automated methods for updating GeneRIFs and the gene-disease mappings. These diseases-gene mappings are provided by three sub-components: (1) associating genes to DO terms using NCBO Annotator [7], (2) a quality control (QC) algorithm for the mapping results and (3) a data aggregation function. NCBO Annotator is a free online text mining tool that integrates a wide range of biomedical ontologies into concept annotation service [7]. The Annotator identifies ontology terms shown in submitted text based on user assigned ontologies and returns descriptions of matched annotations. By taking advantage of hierarchical structure of the ontologies, the Annotator allows to return more general concepts relevant to a specific term by controlling mining parameters. User can programmatically send text mining jobs to the web services provided by the Annotator and retrieve the mining results. The DOAF implemented a program wrapper to automatically submit GeneRIF text to the NCBO Annotator, gather the text mining results, and to map gene-disease relationships based on the results.

Because GeneRIF and DO are constantly improving, the DOAF provides functions for incremental and full updates of the gene-disease mappings. When it is necessary to fully rebuild gene-disease relationships (e.g. the structure or content of DO or GeneRIF has substantially changed), the system can perform a full update of gene-disease mappings to completely rebuild the annotations. An incremental update of mapping adds updated content into the DOAF system and deactivates old mapping results which are no longer contained in the latest DO or GeneRIF. This partial update strategy not only saves computation, but also ensures easy access to the newest disease-gene association information. For reviewing and ensuring high quality annotations, the DOAF implements rule-based QC to check mapping results. For a new mapping result produced by either an incremental or full update, this module computationally checks the result by a Java-implemented heuristic method, which can detect uninformative mappings such as “Disease” (too broad definition) and incorrect mappings such as calcium being mapped to cancer (both of them can be abbreviated as “Ca”). For computational efficiency, we created a mis-mapping repository to prevent re-checking and re-correcting of previous identified errors.

## Web Interface, Usage, and Future Work

The DOAF framework is freely available online (http://doa.nubic.northwestern.edu) and is updated monthly. The DO can be downloaded and viewed or edited with OBO format compatible software. The framework provides an easy and user-friendly web interface to query the annotation mappings either for genes of interests with disease descriptions or diseases of interests with gene names. The query supports gene aliases and synonyms mode. Querying results are shown in a table or a tree style of views. The resulting key terms such gene name can be linked out to an external source and sorted according to hit number or alphabetical order. Users can download the results.

Here is one use case: a user inputs “primary breast cancer” in text field and clicks “To Gene” button to search the relevant genes associated with the input disease along with detailed descriptions. There will be 50 results according to hit GeneRIF entries. One “primary breast cancer” related gene can have multiple GeneRIF entries. For example, the first gene listed in the result table is ADAM17. There is one GeneRIF entry showing the evidences that the gene ADAM17 is “primary breast cancer” related. User can further click each PubMed ID to review the corresponding research article. The first GeneRIF is for PubMed ID 17438092 which is an article published at Clinical Cancer Research in 2007, titled “ADAM-17 expression in breast cancer correlates with variables of tumor progression”. This study investigated the interrelationship of the ADAM17 gene and progression of breast cancer [20]. The query for genes of interest with disease descriptions is shown in similar manner.

Further, the DOAF framework supports a programmatic API and HTTP REST service which enables large-scale and integrative analysis by working with external analytic software or online service platforms. This API is being utilized by third part systems biology platforms, such as the Genomic Regions Enrichment of Annotations Tool [21]. The architecture of DOAF is flexible, reusable, and easily integrates all the functional modules such as DO and NCBO Annotator. Therefore, the disease-gene relationship repository constructed by DOAF can also serve a platform to integrate other types of information such as drug-target and molecular interaction network, which will be ideal for providing an integrative analysis of systems biology. In the future, we will extend DOAF to these fields. GeneRIF also contains gene information for many model species in addition to human. Therefore when the corresponding disease ontologies are built, this framework method can be extended to map disease-gene relationships of additional species.
